# Hazardous impacts of heavy metal pollution on biometric and biochemical composition of pearl oyster *Pinctada radiata* from five sites along Alexandria coast, with reference to its potential health risk assessment

**DOI:** 10.1007/s11356-024-32571-z

**Published:** 2024-02-28

**Authors:** Hala Ahmed Abdel-Mohsen, Mona Mohamed Ismail, Ragia Moussa Moussa

**Affiliations:** 1 Marine Pollution Laboratory, Environment Department, National Institute of Oceanography and Fisheries, NIOF, Cairo, Egypt; 2 Taxonomy and Biodiversity of Aquatic Biota Laboratory, Environment Department, National Institute of Oceanography and Fisheries, NIOF, Cairo, Egypt; 3 Invertebrate Aquaculture Laboratory, Aquaculture Department, National Institute of Oceanography and Fisheries, NIOF, Cairo, Egypt

**Keywords:** *Pinctada radiata*, Metal pollution, Condition index, Morphometry, Antioxidant activities

## Abstract

This study investigated the effect of heavy metals on the pearl oyster *Pinctada radiata* from 5 sites along the coast of Alexandria, with focus on its ecological health and potential risks to human consumption. Pollution results showed that Abu-Qir had the highest Cu and Cd values. Montaza and Eastern Harbor had the highest Fe and Pb values, respectively. Statistically, differences in metal concentrations among study sites were significant (*p* < 0.05). Non-carcinogenic risk (TTHQ) of tested metals and carcinogenic ones of Cd and Pb showed “high risk” on human health by consuming pearl oysters. Morphometric measurements and condition indices were studied to assess growth patterns and health in relation to heavy metals exposure. Key findings showed detectable declines in size and condition index in Eastern Harbor, whereas Abu-Qir recorded the highest values. This condition index performance presented Abu-Qir, Mammora, and Miami as ideal locations for spat collection and oyster rearing, potentially enhancing Egyptian pearl farming. Average values of spatial proximate contents of pearl oyster showed that it was rich in proteins (33.07–58.52%) with low fat content (1.39–1.87%) and carbohydrates (9.72–17.63%). Biochemical composition of pearl oyster demonstrated its high nutritional value which supported its promotion as a functional food for human consumption. The calorie content of pearl oyster was less than 2 Kcal, making this species an alternative source of healthy food to reduce obesity. Regression analysis indicated that Cu, Cd, and Pb had significant effect on 2-diphenyl-1-picrylhydrazyl (DPPH) scavenging activity, calories, vitamins, and pigment content of the collected oysters.

## Introduction

The rayed pearl oyster *Pinctada radiata* (Leach 1814) has an Indo-Pacific origin inhabiting temperate, tropical, and subtropical waters. It was first recorded in the Egyptian Mediterranean waters in 1874 as *Meleagrina* sp. (Monterosato 1878). It shows a widespread distribution in the Mediterranean and European waters with great interest in its considerable value as a food source for human consumption, in addition to their ability to produce exquisite pearls (Theodorou et al. 2019). Bivalve general health condition can be assessed by widely used applicable common tools such as allometric relationship and condition index (CI) reflecting the adaptation ability to environmental conditions (Lim et al. 2014). In addition, measurement of CI could be used to address the stress condition of oyster exposed to heavy metal concentrations as suggested by Aguilar et al. (2012). The prominent ability of oysters to increase its spatial distribution may be enhanced by its peculiar tolerance to chemical contamination that makes it a model organism in ecotoxicological assays reflecting metal pollution stress in marine environment (Cabral et al. 2022). In addition, they have the ability under pollution stress, to exhibit antimicrobial activities (Chan et al. 2021a). Generally, oysters are known to be natural unique efficient bioaccumulators of large amounts of most metals in their body tissues due to their ability of selective accumulation of heavy metal concentrations (Aslam et al. 2020). In fact, they are able to filter up to 1500 L of sea water leading to more than 100 times higher pollutant concentrations in the oyster body than ambient water (Sarma et al. 2013). One of the primary consequences of heavy metal pollution in pearl oysters is the impairment of their physiological processes (Chan and Wang 2019). Heavy metals pollution can negatively affect the reproductive capacity of pearl oysters through interference with the reproductive cycles leading to reduced reproductive success, impaired larval development, and increased mortality rates (Weng and Wang 2019). As pearl oysters are vital components of marine ecosystems, their decline can disrupt the overall biodiversity and ecological stability of coastal areas (Naser 2013). Furthermore, the pearl industry, which heavily relies on the cultivation and harvest of pearl oysters, can experience significant economic losses if pearl oyster populations are compromised due to heavy metals pollution (Orban et al. 2002).

Bioaccumulation of heavy metals in aquatic organisms can have implications on human health and ecosystem (Aslam et al. 2022). The major hazardous impacts of heavy metals bioaccumulation on human health are toxicity to the renal, nervous, and reproductive systems, in addition to mutations and endocrine disruption (Brar et al. 2010). Copper, iron, cadmium, and lead are among the major toxic metals that are bioaccumulated in marine organisms posing a risk to human consumers via food chain (Azizi et al. 2018). The adverse health effects of such toxic and carcinogenic metals have driven attention to the necessity of measuring heavy metals concentrations in different foodstuffs. In that context, some indicative parameters are implemented as efficient tools to assess the potential risks on human health resulting from the consumption of exposed food items to environmental pollution. These parameters include estimated daily intake (EDI), target and total hazardous quotients (THQ and TTHQ), in addition to the assessment of carcinogenic risk (CR).

It is well-known that bivalve mollusks, particularly oysters, are one of the most valued affordable food sources that have high protein content with a nutritional value, vitamins, minerals (Na, K, Ca Mg), essential fatty acids, and amino acid offering benefits to the human body (Biandolino et al. 2020). Biochemical alterations of oysters and mussels are widely used in pollution monitoring studies due to their quick response to aquatic stressors (Sarma et al. 2013). Additionally, oysters have antioxidant, immunological, and antitumor properties which were ascribed to their bioactive contents (Sugesh et al. 2019). Natural antioxidants are critical in assisting endogenous antioxidants in neutralizing oxidative stress (Pachaiyappan et al. 2014). So knowledge of the biochemical content of oysters provides essential information about its nutritional value and the state of the harboring area.

In recent years, many world-wide studies as well as local ones investigated heavy metals bioaccumulation and health risk of different oyster species including pearl oyster. Mok et al. (2015) studied the bioaccumulation of heavy metals in oysters from Korean coast, in addition to their health risk assessment. Chan et al. (2021a, b) observed the impact of pollution stress on the physiology of different shellfish species and their antimicrobial activities as a survival mechanism, depending on species size. Cabral et al. (2022) carried out an experimental bioassay for 10 days on *Crassostrea rhizophorae* oyster, by applying different concentrations of Fe and Mn, either separately or synchronized and observed their effects.

In Egypt, Radwan et al. (2012) examined the ecotoxicological and physiological aspects of *P. radiata* from the Mediterranean Sea. Atia et al. (2018) monitored heavy metal residue in some shellfish including oysters from fish markets in Ismailia Governorate, Egypt. Eltanani (2021) observed heavy metals in some shellfish including oysters from fish markets in Kalyobia Governorate, Egypt.

Therefore, the present study was carried out on *P. radiata* as a candidate pearl oyster species for Egyptian food and pearl production in order to (1) determine and evaluate major ions quotient index (MIQ) and the concentration of four heavy metals (Cu, Fe, Cd, Pb) in the soft edible portion (EP) of oyster from 5 study sites along Alexandria Coast, Egypt; (2) address the human health risk of the tested heavy metals; (3) assess the impact of pollution on condition indices and allometric relationship; and (4) investigate oysters’ proximate composition, pigments, vitamins (C and E) content, and antioxidant activity of oysters as affected by heavy metals contamination. The present study could be of importance in providing guidelines of the ongoing safety monitoring of oyster natural resources and human public health risk assessment. Such knowledge would be crucial indicator of oyster contamination condition and would explore the most polluted areas along the coast of Alexandria, Egypt.

## Materials and methods

### Study sites

**Fig. 1:**
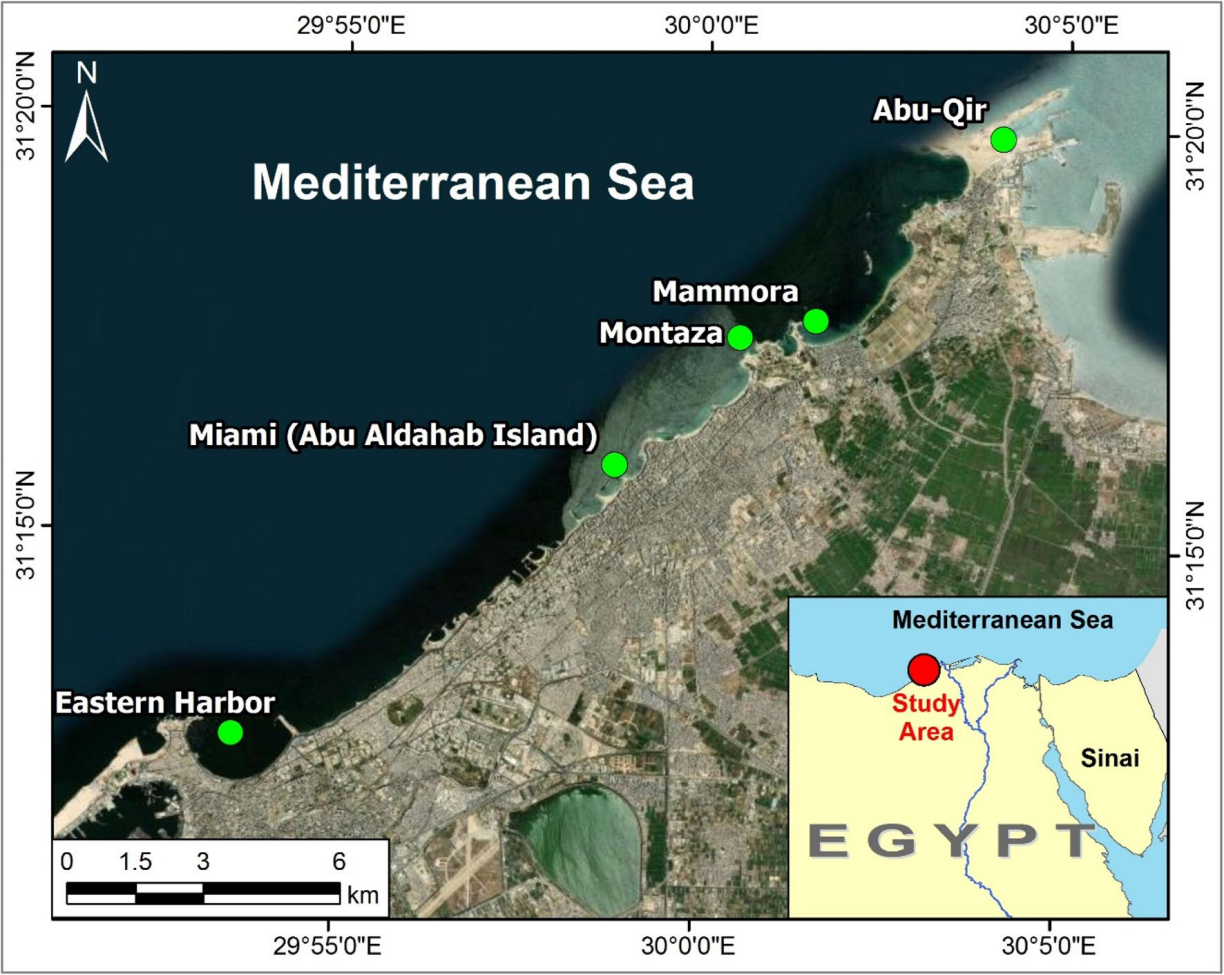
Map showing the 5 study sites along Alexandria coast, Egypt

Samples of the present study species *P. radiata* were collected from 5 study sites lying along the coast of Alexandria. From east to west, the study sites are Abu-Qir (31.331809, 30.067912), Mammora (31.294687, 30.025662), Montaza (31.291029, 30.008257), Miami (31.26514, 29.97998), and Eastern Harbor (31.20985, 29.89299) (Fig. 1). Abu-Qir is a shallow semi-closed basin that is subjected to several land-based sources, like freshwater from the Rosetta mouth of the Nile, loaded with nutrients. Lake Edku also pours effluents that are carried with heavy metals, pesticide, humic acids, and nutrients into the bay. El-Tabia pumping station pours industrial and domestic wastes into the bay as well. Abu-Qir Bay is also exposed to oil pollution from fishing boats, in addition to the activities of Abu-Qir Fertilizers Company and Abu-Qir Electrical Power Station (Shams El-Din and Dorgham 2007). The study sites Montaza, Mammora, and Miami are among 35 beaches that lie along the 24 km long coast of Alexandria. These beaches used to receive discharge from drainage water of Alexandria. Two decades ago, the discharge was stopped and the only source of pollution in the beaches became the activities of vacationers, during summer and autumn (Alprol et al. 2021). Eastern Harbor is a shallow semicircular basin that had always been the recipient of large volumes of domestic waste water from several outfalls. Nowadays, only one outfall remained which disposes its charge into the south-west bay margin (Ismael and Khadr 2003).

### Collection and preparation of samples

In the collection period of June 2021, a total of 366 samples of *P. radiata* similar sizes were collected from the five study sites. Samplings were performed by SCUBA diving from soft and rocky substrates at 2–4 m depths. After collection, alive oysters were stored in plastic bags with ice and transported to the laboratory. In the laboratory, oyster shells were scrubbed to remove mud and epizoic organisms and subsequently rinsed with seawater. The identification of *Pinctada radiata* was done according to taxonomic features mentioned by Scuderi et al. (2019).

### Length–weight relationship and condition index

#### Morphometric measurements

**Fig. 2:**
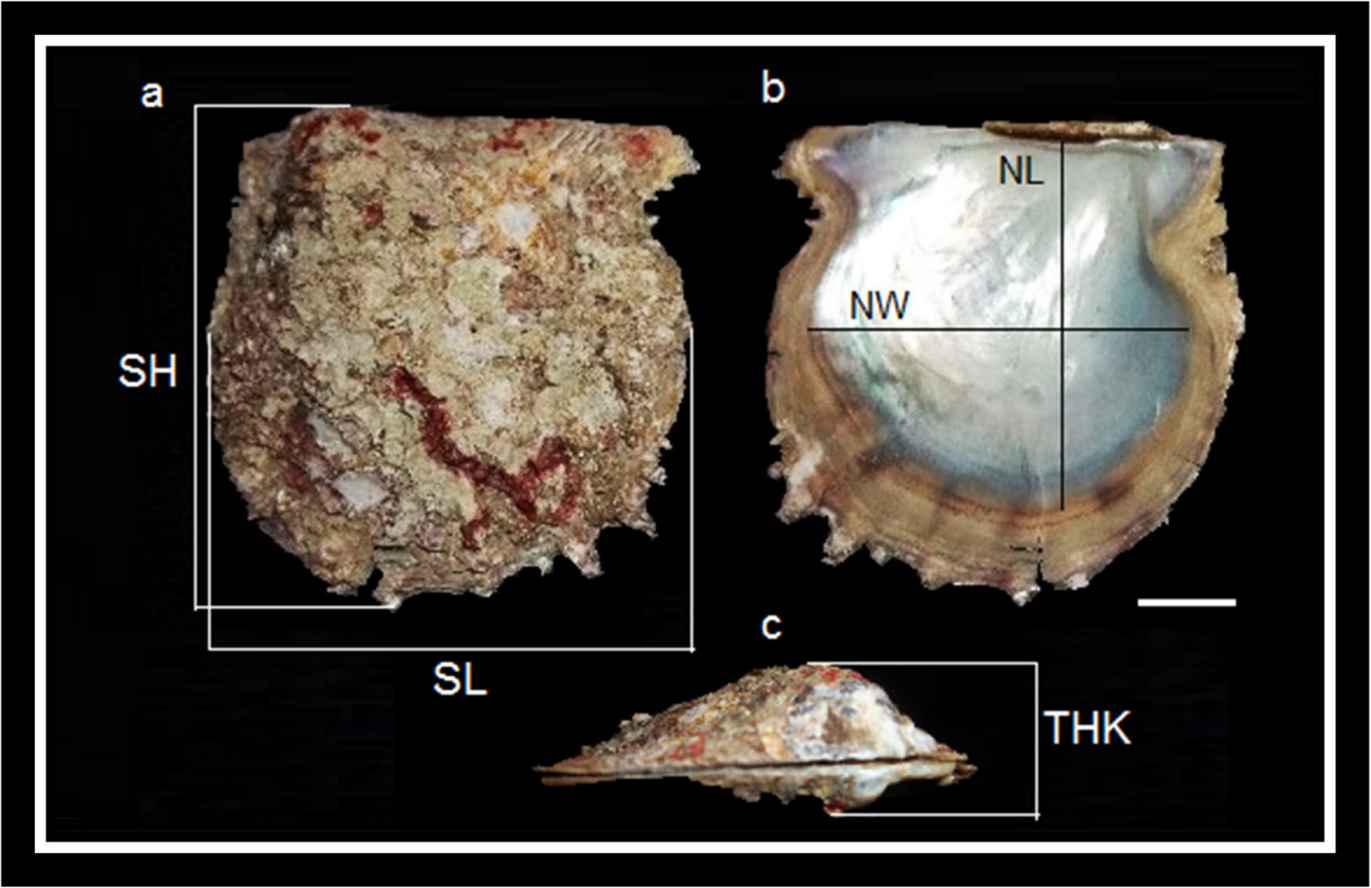
Morphometric measurements of pearl oyster *Pinctada radiata* on **a** dorsal view, **b** internal shell view, and **c** lateral view. SL, shell length; SH, shell height; THK, shell thickness; NL, nacre length; NW, nacre width. Scale bar = 1 cm

For each specimen, the following measurements were taken using a vernier caliper for their biometrical parameters, namely, shell length (SL) (maximum measure along the anterior–posterior axis), shell height (SH) (maximum lateral axis), thickness (THK) (depth of maximum longitudinal axis), nacre length (NL) (maximum nacre lateral axis), and nacre width (NW) (maximum measure along the nacre anterior–posterior axis) (Fig. 2). The samples were then weighed, opened by cutting the adductor muscle with a scalpel, and the wet flesh weight and shell weight were noted. The dry mass (DM) and dry shell mass (DSM) were determined by oven drying of the wet tissues and shells for 48 h at 60 °C, respectively.

#### Length–weight relationship

The relationship between shell height (SH) and total weight (TW) was described by the following equation (Pauly 1983):

$${\text{TW}}\;=\;a{\;{\text{SH}\;}}^{b}$$

This equation can be expressed in its linearized form (Parsons 1988):

$$\text{Log}\;\text{TW}=\text{Log}\;a\;+\;b\;L\mathrm o\mathrm g\;\mathrm S\mathrm H$$

where *a* is the intercept (initial growth coefficient) and *b* is the slope (relative growth rates of the variables).

The degree of association between variables was calculated by the correlation coefficient (*r*). The oyster attains isometric growth when *b* = 3; negative allometric growth when *b* < 3 or positive allometric growth when *b* > 3 (Elamin and Elamin 2014). When the slope *b* is equal to 1, relationships between linear variables (measurements) are isometric.

#### Condition index

Condition indices (CI) were used to characterize the apparent health and quality of the biological entity. Four condition indices taken into account as follows:

I. CI_1_ = DM / DSM (Walne 1976)
II. CI_2_ = DM / [(NL × NW) / 2] × 10,000 (Meyer et al. 1998)
III. AFNOR CI_3_ = (Flesh weight / Total weight) × 100 (Crosby and Gale 1990)
IV. CI_4_ = DM / SH.^*b*^ (Acharya and Dwivedi 1985)

where *b* is the slope in [log DM = *a* + *b* (log SH)].

### Distribution of heavy metals in EP of P. radiata

Soft edible portion (EP) of pearl oyster *P. radiata* (flesh and viscera) from at least 3 to 10 specimens (*N* =  ± 10) were separated and dried in oven at 70 °C and then digested with concentrated nitric acid (Merck, Germany) at room temperature for heavy metals analyses (Idera et al. 2015). After digestion, the samples were diluted to 25 mL with deionized water and then were filtered by Whatman No. 1 into clean, dry polyethylene bottles until they were used for analyses. Heavy metals (Cu, Fe, Cd, and Pb) concentrations were measured by Perkin Elmer 2830 Flame Atomic Absorption Spectrophotometer at wave lengths of 324.7 nm for Cu, 248.3 nm for Fe, 228.8 nm for Cd, and 220.0 nm for Pb, with detection limits of 0.008 μg/g, 0.007 μg/g, 0.005 μg/g, and 0.009 μg/g, respectively.

### Quality control and assurance

In the present study, care was taken to use all reagents of analytical grade and of low trace element content. All procedures of metal analyses were carried out in a precautionary manner, i.e., all glassware and Teflon vessels were previously soaked overnight with 20% HNO_3_ and then rinsed with deionized water. For heavy metal analyses, stock standard solutions (Merck, Germany) of 1000 mg/L, were first prepared and then diluted with deionized water. Concentrations of measured metals were detected according to the described standard conditions in the instrument manual. Triplicate detections were performed for each sample by direct aspiration into air/acetylene flame of the instrument. The precision of heavy metal detection was performed by a reference material, International Atomic Energy Agency, IAEA-405.

### Health risk assessment of P. radiata

#### Estimated daily intake (EDI)

Estimated daily intake (EDI) was calculated according to the method described by the Human Health Evaluation Manual of US Environmental Protection Agency (USEPA 2010) as follows:

$$\mathrm{EDI }({\text{mg}}/{\text{kg}}/{\text{day}})=\frac{C\times {\text{FIR}}\times {\text{EF}}\times {\text{ED}}\times {\text{CF}}}{{\text{AT}}\times {\text{BW}}}$$

where *C* is the concentration of tested metals (mg/kg), FIR is the tested food ingestion rate of bivalves in Egypt, which was estimated as 48.57 g/day for adults (FAO 2003), EF is the exposure frequency (365 days), ED is the exposure duration (60 years), and CF is the conversion factor in kg/gm, whereas AT is averaging time (21,900 days) and BW is the average body weight of Egyptian adults, which was estimated as 70 kg according to El Said et al. (2021).

#### Non-carcinogenic risk

In order to assess the potential non-carcinogenic risks on human health by consuming polluted food as oysters, target hazard quotient (THQ) was estimated for each pollutant. Additionally, total target hazard quotient (TTHQ) was calculated as the sum of THQ of all pollutants according to El Said et al. (2021), as follows:

$${\text{THQ}}=\frac{{\text{EDI}}}{{\text{RFD}}}$$

$${\text{TTHQ}}={\sum }_{1}^{n}{\text{THQ}}n$$

where RFD is the reference oral dose of each pollutant estimated as Cu 4.0 × 10^−2^, Fe 0.7, Cd 5.0 × 10^−4^, and Pb 3.5 × 10^−3^ mg/kg/day according to USEPA (2000) and *n* is the number of tested heavy metals, which in the present study = 4 heavy metals.

#### Carcinogenic risk (CR)

Carcinogenic risk (CR) indicates the possibility of an individual to develop cancer due to the continuous augmenting exposure to potential carcinogens. It was calculated according to the following equation mentioned by USPEA (2000):

$${\text{CR}}={\text{CSF}}\times {\text{EDI}}$$

where CSF is the carcinogenic slope factor. CSF is only available for Cd and Pb and is estimated to be 6.3 (mg/kg/day)^−1^ for Cd and 0.0085 (mg/kg/day)^−1^ for Pb, respectively (USEPA 2000).

### Major ions quotient (MIQ)

For major ions quotient (MIQ) calculations, sodium and potassium concentrations were measured in the soft portion of pearl oyster (EP) by means of Flame 139 Photometer PFP, JENWAY 7. Calcium and magnesium concentrations were determined by the EDTA titration method using murexide and eriochrome black T indicators, respectively (APHA 1999). It was calculated according to the equation mentioned by El Said et al. (2021) as follows:

$${\text{MIQ}}=\frac{\left[\left({{\text{Ca}}}^{+2}\right)\right]+[\left({{\text{Na}}}^{+1}\right)]}{\left[\left({{\text{Mg}}}^{+2}\right)\right]+[\left({{\text{K}}}^{+1}\right)]}$$

### Proximate contents

Oysters’ total proteins were estimated by using Biuret method (David and Hazel 1993). Bovine serum albumin was utilized as a protein standard. The concentration of proteins was detected using the following equation:

$$Y=C\times VM$$

where *Y* is the proteins yield (mg/g), *C* is the protein concentration (mg/mL), *V* is the volume of the proteins extract (5 mL), and *M* is the dry weight of oyster (g).

Total carbohydrate content of the collected oyster samples was assessed spectrophotometrically at 490 nm according to Dubois et al. (1956) using D glucose as a standard. The total lipid content was determined by Folch method (Nielsen 2010) using methanol-chloroform mixture. The results were expressed as a percentage of the oysters’ DW.

#### Pigment contents

Carotenoid content was extracted in acetone (90%) and detected according to the method of Zheng et al. (2012) using the following formula:

$$\mathrm{Carotenoids\;mg}/{\text{g}}\;=\;\frac{{A}_{480}\times\;V\times\;n\times 1000}{E\times\;wt}$$

where *A*_480_ is the absorbance at 480 nm, *V* is the volume of the extracting solution (mL), *n* is the dilution number, 1000 is a constant, *E* is the extinction coefficient (mean value A^1%^_1cm_2500 of colored carotenoids), and *wt* is the mass (g) of the sample.

Fucoxanthin content in the tested *P. radiata* samples was extracted in DMSO:water (4:1, v/v) and estimated µg/g dry weight by using the following equation (Osório et al. 2020):

$$\mathrm{Fucoxanthin\;}(\mathrm{\mu\;g}/\mathrm{g\;DW})\;=\;7.69\times\;({\text{A}}480-{\text{A}}750)-5.55\times [({\text{A}}631-{\text{A}}750)+({\text{A}}582-{\text{A}}750)-0.297\times ({\text{A}}665-{\text{A}}750)]-0.377\times ({\text{A}}665-{\text{A}}750)$$

β-Carotene was determined following the method of Loganathan et al. (2010). The absorbance of the filtrate was measured at 453, 505, and 663 nm. The β-carotene concentration was calculated according to the referred equation:

$$\upbeta\;-\mathrm{carotene\;}({\text{mg}}/100{\text{g}})=0.216\times {\text{A}}663-0.304\times {\text{A}}505+0.452\times {\text{A}}453$$

#### Content of vitamins C and E

Following spectrophotometric methods, vitamin C (ascorbic acid) and vitamin E content from oyster samples were detected according to Saeed et al. (2018) and they were expressed as mg AA/100 g dry weight and mg α-tocopherol/100 g DW, respectively.

### Nutritional value

The total calories and energy contents were determined by the following equations according to the method of Brett and Groves (1979):

$$\mathrm{Calories\;}({\text{kcal}}/100\mathrm{\;g})=4\times {\text{proteins}}(\mathrm{\%})+9\times\;{\text{lipids}}(\mathrm{\%})+4\times \mathrm{carbohydrates\;}(\mathrm{\%})$$

$$\mathrm{Energy\;}({\text{kJ}}/\mathrm{g\;DW})\;=\;36.42\times\;\mathrm{Lipid\;}(\mathrm{\%})+23.86\times\;\mathrm{proteins\;}(\mathrm{\%})\;+\;17.16\times\;\mathrm{Carbohydrates\;}(\mathrm{\%})$$

### Antioxidant activity assay

#### DPPH radical scavenging capacity

The ability of methanolic extract of the collected *P. radiata* samples to scavenge the DPPH (1,1-diphenyl-2-picrylhydrazyl) radical was investigated using the technique of Yen and Chen (1995). The antioxidant activity was measured at 517 nm and estimated by the following equation as an inhibition percentage of DPPH radical (% inhibition):

$$\mathrm{\%\;inhibition}=\frac{Ac-As}{Ac}\times\;100$$

where *Ac* is the absorbance of the control and *As* is the absorbance of the sample.

### Statistical analyses

Paired sample *t*-test was used to analyze the coefficient of correlation of the studied biometric parameters. The condition indices recorded from the five study sites were compared using ANOVA and a pairwise multiple comparison test. Post hoc analysis was conducted with pairwise Duncan’s multiple range test. The statistical analysis of two-way analysis of variance (ANOVA) and Kruskal–Wallis tests were conducted using SPSS software, version 27. The latter test and software were also used to compare and describe the statistical differences among the average concentrations of heavy metals recorded at the 5 study sites at a confidence limit of 95% (*p* < 0.05). A one-way ANOVA analysis was utilized to compare differences in the means of the estimated biochemical parameters of the 10 specimens from each of the 5 study sites, at *p* < 0.05. The statistical analyses of multiple stepwise regressions for the data were done using STATISTICA software, version 12. Twelve studied variables in the collected oysters (10 specimens) from each site were used to build the following equation (significance of *p* < 0.05 and multiple regression coefficients *R*):

1 $$y={\beta }_{0}+{\beta }_{1}A+{\beta }_{2}B+{\beta }_{3}C+\cdots$$

where *y* is the selected variable in oysters; *β*_0_ is a constant; *β*_1_, *β*_2_, and *β*_3_ are the regression coefficients; and *A*, *B*, and *C* are the detected parameters.

## Results and discussion

### Distribution of heavy metals in EP of *P. radiata*

In the present study, average concentration values (± S.D.) of four heavy metals (Cu, Fe, Cd, Pb) were detected in EP of the pearl oyster *P. radiata* (Table 1). For Cu, recorded averages at Abu-Qir, Mammora, Montaza, Miami, and Eastern Harbor were 10.45 ± 2.6 µg/g, 7.44 ± 0.88 µg/g, 4.58 ± 0.94 µg/g, 7.57 ± 1.12 µg/g, and 1.94 ± 0.27 µg/g, respectively. Fe average values were 188 ± 3.54 µg/g, 211 ± 0.83 µg/g, 278 ± 3.54 µg/g, 198 ± 1.64 µg/g, and 236.9 ± 3.09 µg/g, respectively. Notably, three regions had null Cd concentration, namely, Mammora, Miami, and Eastern Harbor, while Abu-Qir and Montaza had average values of 1.99 ± 1.12 µg/g and 0.07 ± 0.19 µg/g, respectively. Pb had averages of 7.84 ± 0.8 µg/g, 2.57 ± 0.31 µg/g, 14.13 ± 1.47 µg/g, 15.06 ± 1.56 µg/g, and 22.32 ± 1.95 µg/g at the five study sites, respectively. These averages had a descending order of Fe (at Montaza) ˃ Pb (at Eastern Harbor) ˃ Cu (at Abu-Qir) ˃ Cd (at Montaza). All study sites, remarkably, had a similar increasing and decreasing attitude order of tested heavy metal values. According to Kruskal–Wallis test, there were significant differences among mean values of tested metal concentrations (*p* < 0.05) and according to the mean value were in favor of Abu-Qir (Cu and Cd), Montaza (Fe), and Eastern Harbor (Pb) (Table 1).

**Table 1:** Average concentration values (± S.D.), maximum (Max.), and minimum (Min.) of heavy metals in pearl oyster *Pinctada radiata* from the 5 study sites along Alexandria coast, Egypt (number of samples *N* = 10)

Study sites	Heavy metals			
	Cu (µg/g)	Fe (µg/g)	Cd (µg/g)	Pb (µg/g)
*Abu-Qir*				
Min	8.75	186	0	6.99
Max	13.34	191	2.56	8.58
Average	10.45^*a*^	188^*b*^	1.99^*a*^	7.84
S.D	± 2.6	± 3.54	1.12	± 0.8
*Mammora*				
Min	6.95	210.5	0	2.26
Max	8.45	211.7	0	2.88
Average	7.44	211	0^*b*^	2.57^*b*^
S.D	± 0.88	± 0.83	0	± 0.31
*Montaza*				
Min	3.43	276	0	12.49
Max	5.73	281	2.5	15.78
Average	4.58	278^*a*^	0.07	14.13
S.D	± 0.94	± 3.54	0.19	± 1.47
*Miami*				
Min	6.54	197	0	13.15
Max	8.76	199.3	0	16.96
Average	7.57	198	0^*b*^	15.06
S.D	± 1.12	± 1.64	0	± 1.56
*Eastern Harbor*				
Min	1.06	234.7	0	20.08
Max	2.97	239	0	23.58
Average	1.94^*b*^	236.9	0^*b*^	22.32^*a*^
S.D	± 0.97	± 3.09	0	± 1.95

^*a*^Denotes highest average values among the study sites

^*b*^Denotes lowest average values among the study sites

In general, heavy metal pollution of aquatic ecosystems is a universal problem that was discussed by many authors, particularly its impact on various oyster species during the last and the present decade. For example, Radwan et al. (2012) marked the influence of heavy metal bioaccumulation on some biochemical parameters (glucose, triglycerides, creatinine, uric acid, and total proteins) of *P. radiata* from different stations along Alexandria Coast. Authors assured the deleterious heavy metals impact on the measured parameters, even at low concentrations. According to Chan et al. (2021a), pollution stress could induce *Magallana bilineata* and *M. cuttackensis* oysters to exhibit antibacterial and antifungal activities as a self-defense mechanism. Bioaccumulation of different heavy metals in *Crassostrea gigas* seemed to be size-dependent; while the maximum Pb levels occurred in oysters of length > 5 cm, highest Cd, Cr, Cu, and Zn levels were in oysters of length from 3 to 5 cm (Liu et al. 2021). In the present study, though the same oyster class-size were examined, variable concentrations of detected heavy metals were reported. It is worth mentioning that in some countries contamination levels are still under control. According to Mok et al. (2015), levels of heavy metal bioaccumulation in oysters from Korean Coast were below standards set by many countries. Consequently, consuming oysters from these sites would not pose any threat to human health.

Copper is one of the essential metals to living organisms; however, excessive amounts of which may cause non-carcinogenic effects on humans such as liver damaging (Cao et al. 2010). Presently, the lowest Cu value was recorded at Eastern Harbor (1.06 µg/g) and the highest at Abu-Qir (13.34 µg/g). The Egyptian Organization for Standardization (EOS 2010) set the maximum permissible limits (MPL) for Cu to be 5 mg/kg. Accordingly, except for Eastern Harbor, study species from all other study sites exceeded the set limits. However, these relatively high Cu concentrations were still not exceeding 120 µg/g which was the level set by USEPA (2000). Low Cu values of Eastern Harbor coincided with those recoded by Eltanani (2021) examining heavy metal levels such as Cu in different shellfishes including oysters collected from fish markets in Kalyobia Governorate, Egypt.

Iron is an essential heavy metal which has significant contributions in many physiological processes, especially respiration, growth, reproduction, and metabolism of shellfishes (El Said et al. 2021). Fe recorded exceptionally high concentration levels in EP of pearl oyster among the four tested heavy metals at all study sites. The lowest Fe value was recorded at Abu-Qir (186 µg/g) and the highest at Montaza (281 µg/g), which could be due to the anthropogenic activities at this site.

Many studies measured the concentration of Fe in different oyster species and detected variable values. Souza et al. (2011) detected a value of 346 µg/g of Fe in *Crassostrea rhizophorae* (Bahia, Brazil). Lino et al. (2016) found 104–477 µg/g of Fe in *Perna perna* (Rio de Janeiro, Brazil). According to Campolim et al. (2017), Fe range was 430–770 µg/g in specimens of *P. perna* (São Paulo, Brazil). Specimens of *C. rhizophorae* collected in Vitória Bay (Brazil) presented approximate concentrations of 1000 µg/g of Fe in their tissues (Vieira et al. 2021). Cabral et al. (2022) experimentally applied higher concentrations of Fe and Mn to *C. rhizophorae* for 10 days resulting in remarkable accumulation of Fe reaching from 2570 to 5220 µg/g. Iron and manganese severely affected *C. rhizophorae* health with a significant damage to the genetic material and enzymatic changes.

Cadmium is a toxic non-essential heavy metal, even at minute concentrations, and is additionally regarded as a probable carcinogen (Kabata-Pendias 2011). Samples collected from Mammora, Miami, and Eastern Harbor were void of Cd. On the other hand, samples from Abu-Qir and Montaza recorded a relatively high Cd values (2.56 µg/g and 2.5 µg/g, respectively) which could be a result of Cd transfer from sediment to the present species. According to Masoud et al. (2012), sediment erosion at Montaza could be the reason for the increased level of Cd in sediment. Pearl oyster is a sedentary species and Cd could have easily been transferred into the animal tissue from sediment. Notably, the highest values recorded in specimens of Abu-Qir and Montaza exceeded the MPL for Cd which is equal to 0.05 mg/kg wet weight (EOS 2010). Similar high Cd values in shellfish flesh were also observed by Eltanani (2021).

Lead is a non-essential highly toxic metal that can cause deep harm to the nervous system and to the urinary system (Gercia-Leston et al. 2010). Currently, the lowest Pb value was recorded at Mammora (2.26 µg/g) and the highest at Eastern Harbor (23.58 µg/g). It is worth mentioning that all values in all study sites were exceedingly higher than MPL set by EOS (2010) for Pb (0.3 mg/kg). Apparently, these values could comprise a high threat to all living organisms in such sites. On the other hand, relatively lower Pb values were recently recorded by Atia et al. (2018) measuring heavy metal residuals in shellfish including other types of oysters. Inter-species accumulation attitude differences could be dependent on the various species-specific types of intra-xenobiotic metabolizing enzymes (Darwish et al. 2019).

### Health risk assessment

#### Estimated daily intake (EDI)

For assessing health risk of *P. radiata* to humans, estimated daily intake (EDI), target hazard quotient (THQ), and total target hazard quotient (TTHQ) values of tested heavy metals were calculated (Table 2). Notably, all EDI values of Cu and Fe in pearl oysters at all study sites were below their reference oral dose (RFD) values: 4.0 × 10^−2^ and 0.7 mg/kg/day, respectively (USEPA 2000). Except for Abu-Qir, all EDI values of Cd were below its RFD (5.0 × 10^−4^ mg/kg/day). Remarkably, Pb EDI values in all study sites were above its RFD value of 3.5 × 10^−3^ mg/kg/day (USEPA 2000). Accordingly, we could consider that consuming pearl oysters from any of the present study sites could be of risk to human populations there. In contrast, lead and Cd dietary intakes by Vietnamese population from shellfish were lower than the provisional tolerable weekly intakes and thus were of safe to consume (Nguyen et al. 2014).

**Table 2:** Values of estimated daily intake (EDI) in mg/kg/day, target hazard quotient (THQ), and total target hazard quotient (TTHQ) of tested heavy metals in pearl oyster *Pinctada radiata* from the 5 study sites along Alexandria coast, Egypt

Study sites	Cu		Fe		Cd		Pb		TTHQ
	EDI	THQ	EDI	THQ	EDI	THQ	EDI	THQ	
*Abu-Qir*	0.007	0.175	0.124	0.177	0.001	2	0.005	1.429	3.781
*Mammora*	0.005	0.125	0.14	0.2	0	0	0.01	2.857	3.182
*Montaza*	0.003	0.075	0.177	0.253	0.0001	0.2	0.01	2.857	3.385
*Miami*	0.005	0.125	0.13	0.186	0	0	0.01	2.857	3.168
*Eastern Harbor*	0.001	0.025	0.16	0.229	0	0	0.02	5.714	5.968

#### Non-carcinogenic risk

THQ was calculated to evaluate the non-carcinogenic effects of the tested metals (Table 2). According to USEPA (2000) standards, THQ are categorized into six classes: HQ < 1 indicating “no health risk”; 1 < HQ < 1.5 indicating “low health risk”; 1.5 < HQ < 2 indicating “medium–low health risk”; 2 < HQ < 2.5 representing “medium risk”; 2.5 < HQ < 3 signaling “next higher risk”; and 3 < HQ denoting “high risk.”

As noticed, THQ values recorded for Cu and Fe lied under the first class (< 1), whereas Cd level at Abu-Qir could only pose medium risk on consumption. As for Pb, while its value at Abu-Qir (1.429) represented the “low health risk” category, levels at three other study sites (Mammora, Montaza, and Miami) belonged to the “next higher risk” category. Remarkably, level of Pb in EP of pearl oysters from Eastern Harbor comprised “high risk” of developing non-carcinogenic symptoms on human health since its value exceeded 3 (5.714). Cd non-carcinogenic severe exposure risk was documented to result in pulmonary effects such as bronchiolitis, emphysema, and alveolitis (Kabata-Pendias 2011). It could also result in bone fracture, kidney dysfunction, and hypertension. On the other hand, Pb exposure could cause disturbances in the normal functioning of kidneys, joints, reproductive, and nervous systems (Ogwuegbu and Muhanga 2005).

#### Total target hazard quotient (TTHQ)

Total target hazard quotient (TTHQ) values are shown in Table 2. Values of TTHQ are also divided into six categories according to the standards of USEPA (2000). All recorded TTHQ values of tested heavy metals exceeded 3 and accordingly belonged to the “high risk” category on human health by consuming pearl oysters. Similar results were obtained by Atia et al. (2018) detecting heavy metal residuals in flesh of different shellfish including oysters (TTHQ = 10.98). The authors revealed a positive correlation between Cd and Pb and ascribed it to the induction of metallothionein which is an enzyme related to metal detoxification. Similar metal–metal interactions in the present species could have contributed to the high risk rank of *P. radiata* TTHQ.

#### Carcinogenic risk (CR)

Carcinogenic risk (CR) was calculated for two heavy metals only (Cd and Pb), depending on their cancer slope factors (CSF) availability (Table 3). Our results showed that Cd CR values in pearl oyster from Abu-Qir and Montaza study sites (0.0063 and 0.00063, respectively) lied above the acceptable lifetime risk (ALR) of 10^−5^ USEPA (2000), indicating a possibility of developing cancer in humans on the long run. According to USEPA (2000), if CR was above the threshold value of the ALR of 10^−5^, it could indicate a probability of 1:100,000 of an individual developing cancer. Incremental consumption of the pearl oyster from either Mammora, Miami, or Eastern Harbor, however, posed no threat of developing cancer. On the other hand, only Eastern Harbor Pb CR value was above ALF (0.00017), suggesting that humans consuming pearl oysters from this site would be at a high risk of developing cancer on the long run. This result was in accordance with the calculated concentration value of Pb in EP of pearl oysters at Eastern Harbor, which was above the MPL set for Pb (0.3 mg/kg ww), according to EOS (2010).

**Table 3:** Values of cancer slope factor (CFS) of Cd and Pb and the calculated carcinogenic risk (CR) in pearl oyster *Pinctada radiata* from the 5 study sites along Alexandria coast, Egypt

Study sites	CSF (Cd)	CR (Cd)	CSF (Pb)	CR (Pb)
*Abu-Qir*	6.3	0.0063	0.0085	0.0000425
*Mammora*	6.3	0	0.0085	0.000085
*Montaza*	6.3	0.00063	0.0085	0.000085
*Miami*	6.3	0	0.0085	0.000085
*Eastern Harbor*	6.3	0	0.0085	0.00017

Our results clearly showed that Cd and Pb were two risky metals and that Abu-Qir and Eastern Harbor were the most affected and unsuitable ecosystems for pearl oyster *P. radiata.* Swift and serious measures from the authorities should be taken to lessen the levels of highly hazardous metals such as Cd and Pb from exposed sites. From their side, consumers should, at least, soak pearl oysters in water before cooking and eating for some time. Boiling or frying would be perfect in reducing significantly metals’ load within oysters’ flesh. Conversely, barbequing or grilling would only increase the concentration of metals in EP of oysters (Atia et al. 2018).

### Morphometric measurements

Table 4 presents detailed information on the maximum, minimum, mean, and standard deviation values of the studied morphometric parameters. Among the study sites, Abu-Qir, Miami, and Mammora displayed relatively high SH values, although they were still smaller than the largest specimen with 113 mm SH and 144.71 g total weight (TW) reported in Abu-Qir by Moussa in 2013. A decade ago, Moussa conducted a study on *P. radiata* growth patterns affecting pearl production in Abu-Qir, Mammora, and Miami. A comparison of the present results with those of Moussa’s (2013) study revealed a noticeable decline in the mean TW, SH, THK, NL, and NW over the last 10 years. However, the mean of SL showed a considerable increase during the same period, while the NL range remained stable. This decline in size could be attributed to larger oysters being more accessible and harvested, leading to a reduction in the biomass and the number of large-sized oysters in their natural habitat.

**Table 4:** Biometric measurements of pearl oyster *Pinctada radiata* from the 5 study sites along Alexandria coast, Egypt (*N*, number of samples; *X*, mean value; *SD*, standard deviation; *Min*, minimum value; *Max*, maximum value)

Study sites	Variable	*N*	Min	Max	*X*	SD
*Abu-Qir*	SH	77	29	76	57.56	8.08
	SL	85	25	77	56.42	7.87
	THK	81	10	26	20.54	3.367
	NL	51	21	58	44.09	7.57
	NW	51	20	55	40.68	7.25
	TW	51	4.02	69	32.71	11.99
*Mammora*	SH	62	39	75	53.83	6.86
	SL	62	40	69	51.31	6.52
	THK	62	12	26	18.71	2.70
	NL	62	27	49	38.69	4.68
	NW	62	22	48	36.53	4.96
	TW	54	10.69	53.82	24.64	8.99
*Montaza*	SH	67	34	69	47.4	8.35
	SL	67	33	69	46.33	8.42
	THK	66	11	23	16.39	2.75
	NL	67	26	51	36.41	7.75
	NW	67	23	5.5	33.07	6.78
	TW	67	6.15	52.25	17.22	8.98
*Miami*	SH	128	26	75	54.13	7.74
	SL	128	29	65	44.85	5.88
	THK	128	9	24	18.78	2.98
	NL	120	20	55	40.27	6.05
	NW	120	15	56	37.8	6.5
	TW	120	2.48	49.58	25.72	9.26
*Eastern Harbor*	SH	23	36	72	50.4	8.42
	SL	23	33	70	46.6	6.6
	THK	23	11	21	16.84	3.47
	NL	23	26	49	36.53	6.54
	NW	23	22	51	33.76	7.75
	TW	23	6.15	48.64	18.82	10.41

#### Length weight relationship

Length–weight relationship (LWR) is a crucial tool in oyster aquaculture as it offers valuable insights on physiological condition, health, and productivity. It reflects the oysters’ overall well-being and adaptability to their environment (Lim et al. 2013). Understanding the relationship between shell height (SH) and other dimensions can shed light on the pattern of shell growth and dimensional changes (de Paula and Silveira 2009). Consequently, SH emerged as the most suitable biometric descriptor for *P. radiata*, enabling predictions of biomass parameters and the definition of marketable size (Lodola et al. 2013).

**Fig. 3:**
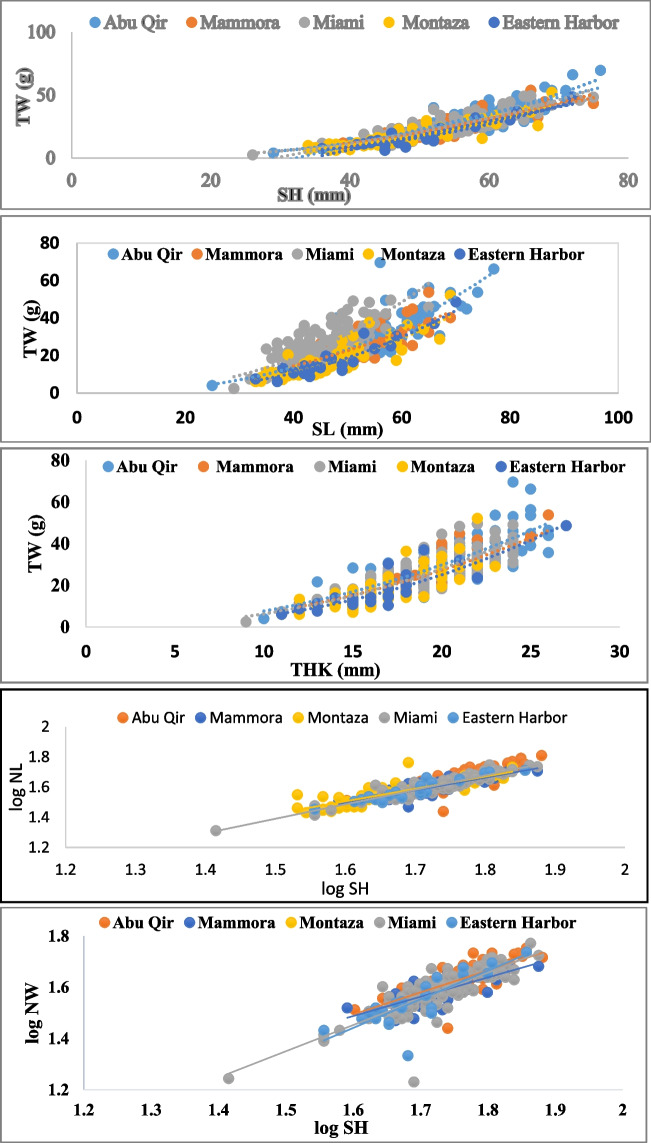
Comparison of relative growth patterns of the different measurements of pearl oyster *Pinctada radiata* from the 5 study sites along Alexandria coast, Egypt

All morphometric relationships represented in Fig. 3 exhibited statistical significance (*p* < 0.01), with coefficient correlation values (*r*) ranging from 0.71 to 0.88 and coefficient determinations values (*R*^2^) ranging from 0.53 to 0.88 (Table 5). Most of the growth patterns displayed a negative correlation between total weight (TW) and other length measurements (SH, SL, and THK) at all sites. The *b* values were significantly less than unity (*p* < 0.001; Student’s *t*-test) (Table 5), indicating that TW increased relatively slower than SH, SL, and THK. These results aligned with previous findings of negative allometry relationships in *P. radiata*, regarding TW-SL (Derbali et al. 2019) and TW-SH (Moutopoulos et al. 2021). Negative allometry has also been reported in various *Pinctada* spp., including *P. fucata*, *P. margaritifera*, and *P. sugillata* (Lim et al. 2020).

**Table 5:** Growth patterns of pearl oyster *Pinctada radiata* from the 5 study sites along Alexandria coast, Egypt (*N*, number of samples; *a, b values*, regression constants; *r*, coefficient correlation, *R*^2^, coefficient determination)

Study sites	Variables	*N*	*A* value	*B* value	Growth pattern	*T*	*R*	*R*^2^
*Abu-Qir*	TW vs. SH	77	0.047	2.575	− ve	33.29	0.860	0.829
	TW vs. SL	85	0.072	2.353	− ve	18.56	0.852	0.726
	TW vs. THK	81	0.339	1.963	− ve	12.41	0.825	0.681
	Log (SH) vs. log (NL)	77	− 0.200	1.051	+ ve	24.30	0.827	0.684
	Log (SH) vs. log (NW)	77	− 0.399	1.118	+ ve	34.31	0.830	0.670
*Mammora*	TW vs. SH	54	0.069	2.333	− ve	43.64	0.839	0.687
	TW vs. SL	62	0.125	2.018	− ve	23.54	0.741	0.548
	TW vs. THK	62	0.305	2.016	− ve	8.20	0.873	0.762
	Log (SH) vs. log (NL)	62	0.154	0.837	− ve	35.51	0.855	0.730
	Log (SH) vs. log (NW)	62	0.278	0.758	− ve	28.27	0.855	0.572
*Montaza*	TW vs. SH	67	0.045	2.555	− ve	30.29	0.754	0.821
	TW vs. SL	67	0.059	2.41	− ve	27.09	0.882	0.777
	TW vs. THK	66	0.170	2.439	− ve	1.63	0.854	0.713
	Log (SH) vs. log (NL)	67	0.190	0.821	− ve	20.86	0.817	0.731
	log (SH) vs. log (NW)	67	− 0.074	1.149	+ ve	31.35	0.757	0.603
*Miami*	TW vs. SH	129	0.055	2.468	− ve	67.50	0.858	0.794
	TW vs. SL	128	0.080	2.362	− ve	20.83	0.733	0.537
	TW vs. THK	128	0.257	2.156	− ve	9.85	0.867	0.751
	Log (SH) vs. log (NL)	120	− 0.042	0.955	− ve	54.36	0.837	0.878
	log (SH) vs. log (NW)	120	− 0.186	1.024	+ ve	19.85	0.777	0.688
*Eastern Harbor*	TW vs. SH	23	0.030	2.767	− ve	8.61	0.709	0.757
	TW vs. SL	23	0.049	2.525	− ve	25.23	0.753	0.832
	TW vs. THK	23	0.211	2.270	− ve	2.43	0.880	0.775
	Log (SH) vs. log (NL)	23	− 0.056	0.964	− ve	28.56	0.762	0.886
	Log (SH) vs. log (NW)	23	− 0.349	1.118	+ ve	31.66	0.799	0.670

Regional differences in oyster allometry have been reported by Soong et al. (1992). Isometric growth of *P*. *radiata* TW-SH was previously reported by Lodola et al. (2013), Bellaaj-Zouari et al. (2012), and Mohammed and Yassin (2003). The observed variability in shell morphology and allometric growth among *P. radiata* populations could be attributed to the differences of the local conditions and environmental factors, such as salinity, wave action, and temperature, which influenced mollusk development and survival (O’Connor and Lawler 2004). Additionally, factors like population density and food resource availability could impact the growth rate (Lodola et al. 2013).

The deposition patterns of nacre layers over implanted nuclei can vary as the oyster grows and its shell changes in shape, potentially influencing the quality and value of the Mabe pearl (Saucedo et al. 1998). In this study, SH exhibited positive allometry (*b* > 1; *p* < 0.05) with NL in Abu-Qir and with NW in all regions except Mammora. This finding indicated that as the shell height of oysters increased, the NL and NW also tended to increase as well. On the other hand, SH-NL exhibited negative allometry in Montaza, Miami, and Eastern Harbor (*b* < 1; *p* < 0.05). However, SH-NW exhibited positive allometry in all study sites (*b* < 1; *p* < 0.05), consistent with findings reported by Moutopoulos et al. (2021) and Lodola et al. (2013) for the relationships SH-NL and SH-NW, respectively.

Size, as body mass or shell size, is considered one of the most significant “internal” factors influenced by metal accumulation in bivalves (Strong and Luoma 1981). However, the interrelationships between metal concentration and body size can vary among different species and metals (Boyden 1974). Notably, despite the fact that Abu-Qir station recorded the highest Cu concentration, its impact on oysters appeared to be less pronounced at this site, where the biometric measurements reached their peak values. Conversely, in Eastern Harbor, the highest Pb concentration value was observed to have an inhibitory effect on growth patterns, aligning with findings reported by Gifford et al. 2006 on *P. imbricata*.

#### Condition index

**Fig. 4:**
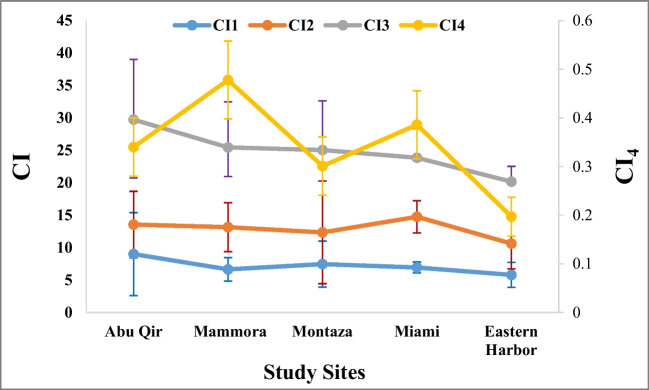
Mean condition index (CI) of pearl oyster *Pinctada radiata* from the 5 study sites along Alexandria coast, Egypt

The condition index (CI) is an economically relevant parameter that effectively describes the commercial quality and physiological state of bivalve mollusks, reflecting their ecophysiological conditions and health in both natural populations and aquaculture settings (Orban et al. 2002). Over the years, various condition indices have been used in both commercial practices and scientific research. In this study, four condition indices were evaluated to obtain data on the stress or wellness condition of *P. radiata* in relation to ambient heavy metal concentrations at five different sampling sites along Alexandria coast. Table 6 displays the results of the two-way ANOVA test, revealing differences between the various condition indices of *P. radiata* at these sampling sites. Significant differences were observed among the various sites for CI_1_, CI_2_, CI_3_, and CI_4_. It is worth noting that the condition indices measured using Walne’s method (1976) reached their maximum in Abu-Qir (ANOVA: *p* < 0.05; post hoc tests). Upon comparing the present findings with previous studies, CI_1_ of *P. radiata* ranged between 5.8 and 9, while *Saccostrea cucullata* exhibited a range of 2.5 to 4.1 (Singh 2019), and *Ostrea edulis* showed a range of 2.6 to 5.11 (Acarli et al. 2011). Moreover, among the various sites studied, CI_2_ exhibited the highest value in Miami, ranging from 10.36 to 13.56 (Fig. 4). This range notably exceeded the values previously reported for *Crassostrea rhizophorae* (3.5–7) as documented by Meyer et al. (1998).

**Table 6:** Two-way tested ANOVA for differences among condition indices of pearl oyster *Pinctada radiata* from the 5 study sites along Alexandria coast, Egypt

CI	Source of variation	SS	df	MS	*F*	*p*
CI_1_	Between regions	57.325	4	14.331	2.983	0.022
	Within regions	581.250	121	4.804		
	Total	638.575	125			
CI_2_	Between regions	262.088	4	65.522	5.297	0.001
	Within regions	1372.932	111	12.369		
	Total	1635.020	115			
CI_3_	Between regions	1.706	4	0.426	81.961	0.000
	Within regions	1.462	281	0.005		
	Total	3.168	285			
CI_4_	Between regions	1073.757	4	268.439	9.476	0.000
	Within regions	7025.556	248	28.329		
	Total	8099.313	252

Regarding the AFNOR CI_3_ measured by Crosby and Gale (1990), significant differences were recorded in the five sampling sites, with higher values observed in Abu-Qir (*p* < 0.01). According to Soletchnik et al. (2001), AFNOR CI_3_ values between 6.5 and 9 correspond to “fine” oysters, while values greater than 9 indicate “spéciale” oysters. Based on this classification, *P. radiata* could be ranked as “spéciale” as AFNOR CI3 values ranged from 20.17 to 29.74 (Fig. 4). In comparison, the previously recorded AFNOR CI values for *Saccostrea cucullata* (Singh 2019) and *Ostrea edulis* (Acarli et al. 2011) were at the ranges of 7.2–12 and 6.65–12, respectively. The present data suggested that the *P. radiata* population along the Alexandria Coast has good quality for consumption.

The condition index (CI_4_) derived from the length–weight relationship (LWR) calculation, using the method of Acharya and Dwivedi (1985), provides a useful comparison of the organism’s weight relative to its length. Kurbah and Bhuyan (2018) stated that CI_4_ values of 1.0 and above indicated better species growth, whereas values less than 1.0 indicated a stress condition. In contrast to the calculated CI_1_, CI_2_, and CI_3_, CI_4_ exhibited lower values in the range of 0.19–0.47 compared to those recorded for *Perna viridis* and *Meretrix meretrix*. This disparity may be attributed to the timing of sampling which coincided with the spawning season. During this period, oysters allocate a substantial amount of energy toward reproduction, leading to a decline in the condition index due to the diversion of energy resources.

It was evident from the study that Abu-Qir recorded the highest values, while Eastern Harbor recorded the lowest values among studied growth patterns and CI. An increase in CI indicated higher levels of organic constituents associated with growth and this increase relied on a delicate balance between food availability, rates of feeding, and rates of catabolism. Conversely, a decline in the CI reflected inadequate nutrition or suboptimal environmental conditions as supported by study of Beninger and Lucas (1984) as observed in Eastern Harbor. Moreover, this CI decrease could be attributed to the inhibitory effect of the highest concentration of Pb. The correlation between higher CI and the production of pearls with superior luster, surface cleanliness, and color highlights their increased value in the pearl market. Consequently, it was highly recommended to collect spat for pearl production and rear the implanted oysters in Abu-Qir, Mammora, and Miami. This approach may enhance the potential for successful pearl cultivation.

### Major ions quotient (MIQ)

**Fig. 5:**
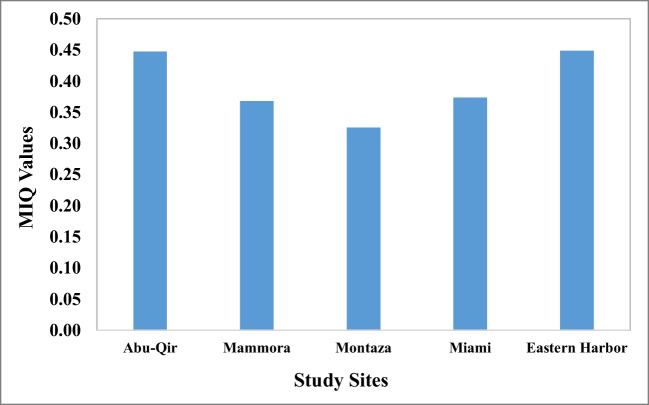
Values of major ions quotient (MIQ) of pearl oyster *Pinctada radiata* from the 5 study sites along Alexandria coast, Egypt

Major ions quotient (MIQ) is one way to indicate the dietary properties of major elements in bivalves as it affects, in turn, element ratios in humans (Csikkel-Szolnoki et al. 2000). Presently after measuring concentration of major ions (data are not shown), MIQ values were calculated on molar bases (Fig. 5). They were lower than that required for humans (0.23–0.45) and could beneficially decrease the ratio existing in human beings on its consumption. MIQ of humans, generally, ranged between 2.5 and 4. However, under ideal conditions, it approximated 1 (Csikkel-Szolnoki et al. 2000). Previous work on oysters’ MIQ are rare; however, the nearest group were recorded by El Said et al. (2021), working on different shrimp species and reported MIQ of 2.9 and 5.3. Higher values than the required human range could pose a threat of developing hypertension, preeclampsia, and heart diseases to consumers Csikkel-Szolnoki et al. (2000). Fortunately, this was not the case in the present study.

### Proximate analysis

**Fig. 6:**
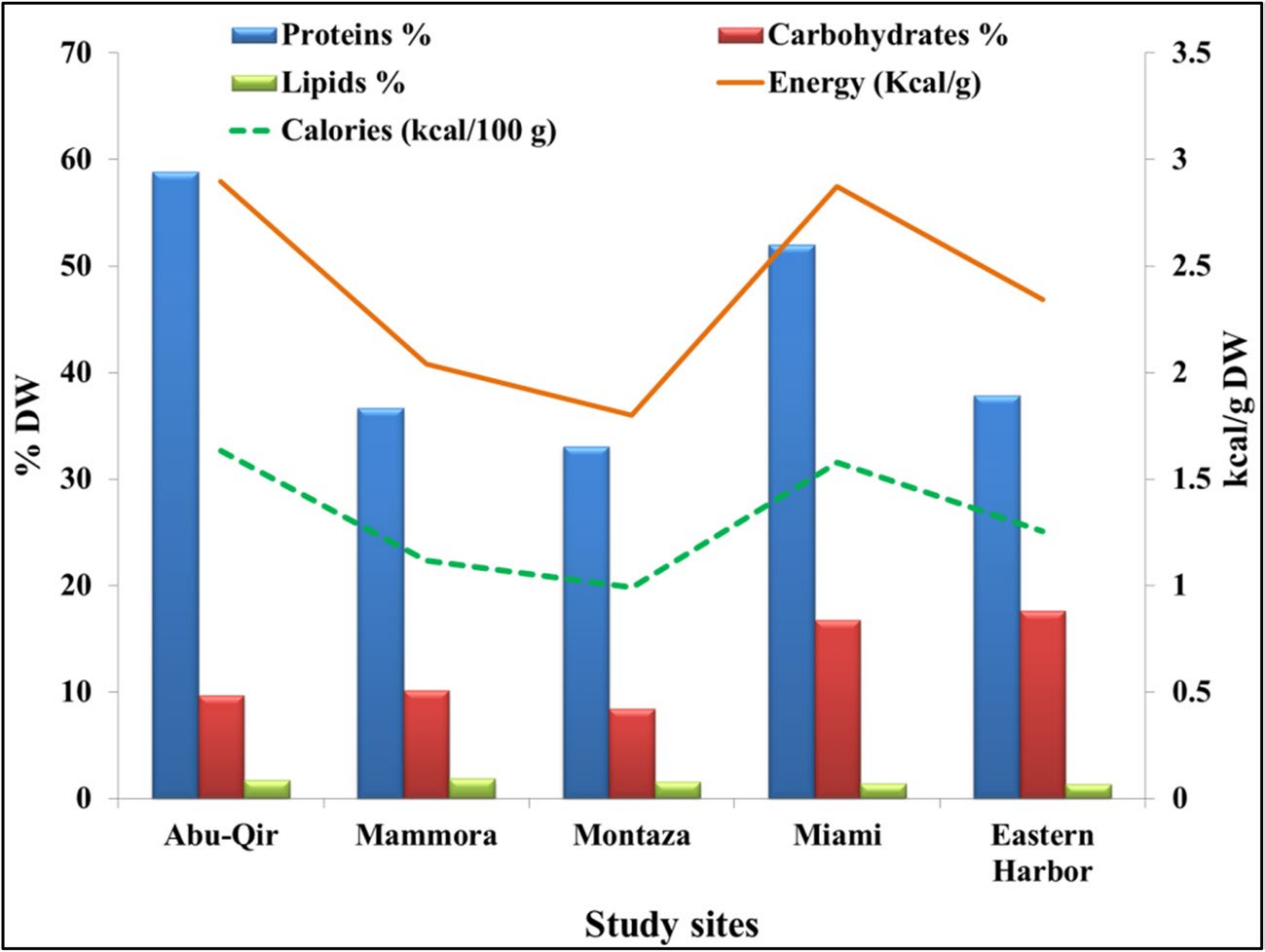
Biochemical composition, energy, and calorie values of pearl oyster *Pinctada radiata* from the 5 study sites along Alexandria coast, Egypt

It is possible to evaluate a species’ edible and nutritional value in terms of energy units by looking at its biochemical composition. Biochemical parameters are recently utilized as a biomarker for anthropogenic pollution due to their quick response to aquatic stressors (Sarma et al. 2013). The proximate composition results of *P. radiata* from different Alexandria coast sites are shown in Fig. 6. These contents serve as a key indicator of their nutritional and physiological health in response to physicochemical parameters of water. Especially, proteins are known to always make up a major portion of the overall oyster biomass, followed by carbohydrates and lipids. This observation aligned with the findings of Theodorou et al. (2021) who detected the protein content in *P. radiata* from Greece represented the main component followed by carbohydrates and fat. Also, Zhu et al. (2018) reported that the proximate contents of the Pacific oyster *Crassostrea gigas* were rich in proteins and low in fat content, whereas the protein concentration in food is crucial in determining its nutritional value and has a significant impact on its market value.

Among the studied samples, the highest protein content was observed in *P. radiata* samples collected from Abu-Qir (58.52%), while it decreased at Montaza (33.07%). Eastern Harbor oyster samples contained the highest amount of crude carbohydrates (17.63% at *p* < 0.05). The total lipid concentrations in oyster samples ranged from 1.39 to 1.87% DW in the samples from Eastern Harbor and Mammora, respectively. These obtained results were in the line of the recent data detected by Makri et al. (2021) who recorded the proximate content of *P. radiata* from CE Aegean Sea (Saronikos and Evoikos Gulf) of the range: 64.33% ± 3.04 for proteins, 11.41% ± 1.43 for fat, and 11.61% ± 3.87 for carbohydrates. Also, the estimated carbohydrate ratio was similar to the edible oysters (*Crassostrea* spp.) (11.3–16.1%) (Minhaz et al. 2020). Lipid content of *P. radiata* (1.09%) from Maden Bay, Turkey, during summer was similar to the estimated ratio while the detected protein ratio (16%) was lower than that observed for the same species (Gokoglu et al. 2006).

In this study, significant variations were observed in all proximate content of the collected specimens between sites, consistent with previous data that linked the variations in the proximate contents of marine seafood to biological parameters such as species, diet, harvest region, catching season, seasonal, and sexual variations (Makri et al. 2021).

**Fig. 7:**
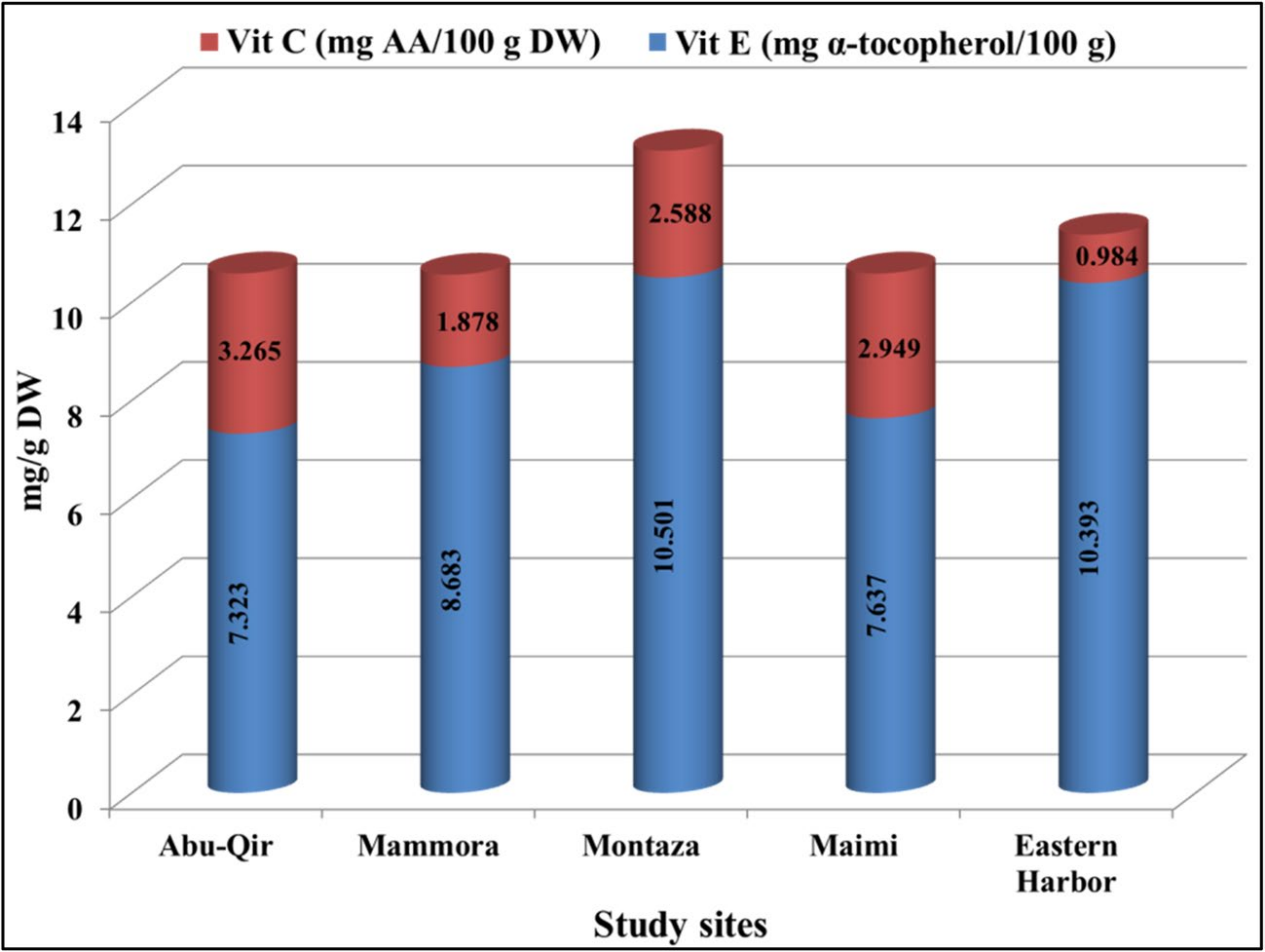
Vitamin E and C content of pearl oyster *Pinctada radiata* from the 5 study sites along Alexandria coast, Egypt

Food quality is correlated with energy and calories, which may be assessed based on the amount of lipid, proteins, and carbohydrate content. The estimated energy content of the tested samples ranged between 1.8 and 2.89 kJ/g DW from Montaza and Abu-Qir, respectively. Similarly, the energy content of *C. gigas* fluctuated between 1.9 and 2.35 kJ/g (Delous-Paoli and Héral 1988). The estimated calorie values had a similar trend of energy contents as shown in Fig. 7. The highest value was observed in the samples (1.63 kcal/100 g DW) from Abu-Qir coast, while the lowest value was detected in Montaza specimens (0.99 kcal/100 g DW). The differences in calorie values of crustaceans were attributed to the total lipid changes (Griffiths 1977). Generally, all of the collected samples had mean energy (2.39 kJ/g DW) and caloric (1.31 kcal) values below the recommended level (2 kcal for women and 2.5 kcal for men) (Benton 2015). Therefore, these oyster species can be regarded as supplemental materials and alternative sources for lowering the risk of obesity.

Carotenoid pigments are widely distributed in plants and algae. Mollusks, such as bivalves, store carotenoids in their body tissues directly from their dietary algae (Maoka et al. 2012)*.* Some of the carotenoids are converted into derivatives of vitamin A in the mollusk tissues (Çolakoğlu et al. 2019).

Significant variation was determined between the total carotenoid contents of the tested samples from different study sites (Table 7). The highest quantity of total carotenoids was detected in samples from Montaza (7.18 mg/g DW) and the lowest content was observed in Eastern Harbor samples (2.64 mg/g DW). The estimated carotenoid content of all tested samples were lower than that detected in *P. fucata* (65–67.6 mg/g) (Zhang et al. 2019), while higher than *Mytilus galloprovincialis* (1.13 ± 0.02 µg/g) and *Crassostrea gigas* (55 µg/g) (Çolakoğlu et al. 2019).

**Table 7:** Pigment contents of pearl oyster *Pinctada radiata* from the 5 study sites along Alexandria coast, Egypt (number of samples *N* = 10)

Study sites	Carotenoid (mg/g DW)	β-Carotene (mg/g DW)	Fucoxanthin (µg/g DW)
*Abu-Qir*	3.195^c^	2.356^b^	0.404^c^
*Mammora*	5.020^b^	1.446^c^	0.517^b^
*Montaza*	7.178^a^	3.092^a^	0.577^b^
*Miami*	5.110^b^	1.764^c^	0.639^a^
*Eastern Harbor*	2.640^c^	1.810^c^	0.766^a^
*F* value	4.82	4.81	39.77

Means with a different superscript letter in the same column were significantly different at *p* ≤ 0.05

Means with the same superscript letter in the same column were insignificantly different at *p* ≤ 0.05

There are several different types of carotenoids found in marine animals, including β-carotene, fucoxanthin, peridinin, diatoxanthin, alloxanthin, and astaxanthin (Zhang et al. 2019). The estimated β-carotene contents (1.446–3.092 mg/g) were higher than that content of different mollusca species (10–960 μg/g), whereas the concentration of carotene in mollusks could be affected by sexual maturity, seasonal change, sources of dietary algae, and whether or not the animal was raised artificially (Zhang et al. 2019).

Very few reports revealed the fucoxanthin content of oyster, although oysters contained considerable amounts of fucoxanthin and its metabolites: fucoxanthinol and halocynthiaxanthin, whereas fucoxanthin and diatoxanthin were identified as major carotenoids in some bivalves (Maoka et al. 2012)*.* Table 7 illustrates significantly high fucoxanthin levels in oyster samples were detected in Eastern Harbor (0.766 µg/g DW) and Miami study sites (0.639 µg/g DW) compared to the other ones.

Marine mollusks are an inexpensive source of proteins with a high biological value as well as vital vitamins and minerals (Idayachandiran et al. 2014). The concentrations of vitamins C and E in all the collected *P. radiata* samples are shown in Fig. 7. Numerous types of seafood that contained high levels of vitamin E are characterized by its anti-inflammatory and antioxidant properties (Niki and Noguchi 2021). Also, vitamin C, often known as ascorbic acid, is renowned for having potent antioxidant properties which prevent oxidative damage to the cell membrane induced by free radicals (Lamidi and Akefe 2017).

The oyster tissue vitamin E content ranged from 10.50 to 7.32 mg α-tocopherol/100 g DW in specimens collected from Montaza and Abu-Qir study sites that was lower than levels of marine bivalve, *Donax cuneatus* (364 mg/100 g DW) (Idayachandiran et al. 2014). Meanwhile, vitamin C content was higher in samples harvested from Abu-Qir (3.26 AA/100 g DW) compared to other sites, also showing statistically significant differences between the collected samples from different stations (*p* < 0.05). The detected vitamin C values of the tested samples were much lower than previously estimated (12.5 mg/g) by Idayachandiran et al. (2014). Similarly, relatively low vitamin C content was detected in fish (1–5 mg/100 g) (Nair and Mathew 2000).

### Antioxidant activity assay

DPPH radical scavenging activity assay has been widely utilized for assessing antioxidant activity, because it can accommodate numerous samples in a brief amount of time and is sensitive enough to detect active components at low concentrations (Pachaiyappan et al. 2014). The ability to remove free radicals from the body may help preventing the oxidative stress linked to myocardial infarction and other cardiovascular disorders (Sugesh et al. 2019).

**Fig. 8:**
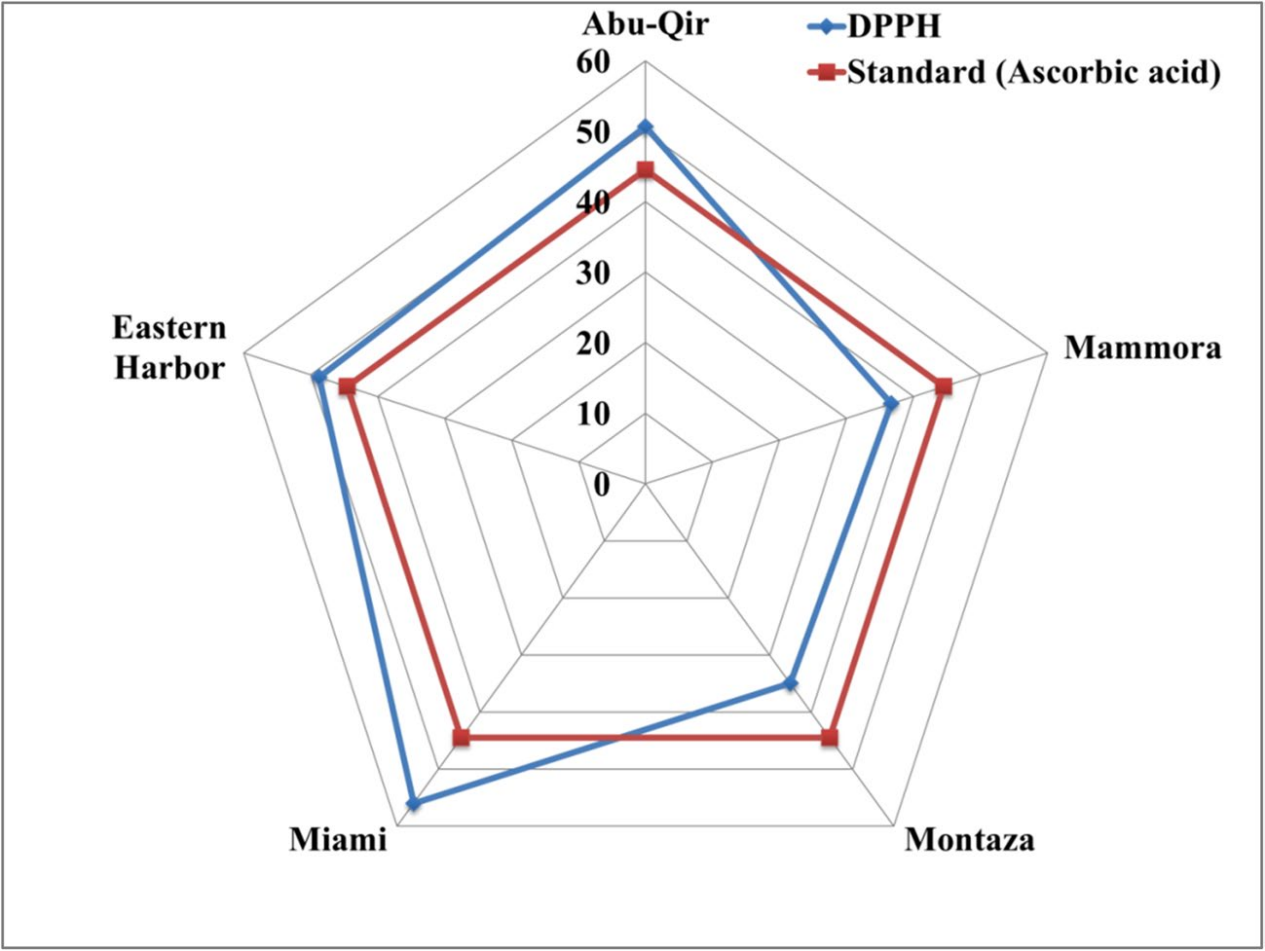
DPPH radical scavenging inhibition activity (%) of pearl oyster *Pinctada radiata* from the 5 study sites along Alexandria coast, Egypt

The percentage scavenging activity of methanolic extract of different oyster samples against DPPH is shown in Fig. 8. Significant differences in DPPH scavenger activity (*p* < 0.05) were observed between the samples collected from different Alexandria sites. Among all the tested samples, the scavenging activity was highest in the samples collected from Miami (56.01%), followed by those from Abu-Qir (50.63%), and lowest for oysters from Montaza (34.93%). These considerable variations in DPPH scavenging abilities of oyster methanolic extracts could have been due to species-specific differences resulting in considerable differences in their chemical contents. The range of DPPH activity of oyster samples (34.93–56.01%) was in agreement with Sugesh et al. (2019) who recorded the DPPH activity of *Meretrix meretrix* to range between 32.45 and 60.45%. However, our findings were lower than the activity of other bivalves such as *M. meretrix* and *Perna viridis* (73.64%), while they were of higher activity than that of gastropod *Turritella attenuata* (23.54%) (Pachaiyappan et al. 2014).

This study indicated that the oyster methanolic extract had the proton-donating activity and could act as free radical inhibitor or scavenger, acting possibly as natural antioxidants where, however, it demanded further research toward purification and mechanisms of action. This result was in line with the observation of Pachaiyappan et al. (2014) who reported that the methanol extract of gastropod and bivalve species could be the better potential source of natural antioxidants.

2 $${\text{DPPH}}\;=\;-18.62\;+\;0.77\mathrm{\;Proteins}\;+\;1.342\mathrm{\;Carbohydrates}\;+\;0.44\mathrm{\;Carotenoid}\;+\;1.29\mathrm{\;Vit}.\mathrm{\;E}$$

Antioxidant effects of the oyster samples on DPPH radical scavenging activity could be related to their content especially total carbohydrates, proteins, carotenoid, and vitamin E as shown in the previous Eq. 2. Polysaccharide content of the bivalve *Mactra veneriformis* might be a significant factor to the antioxidant ability as shown by Wang et al. (2016). Additionally, proteins have great potential as food additives for antioxidants because they can prevent lipid oxidation via a variety of mechanisms, including the inactivation of reactive oxygen species (ROS), the scavenging of free radicals, the reduction of hydroperoxides, the chelation of prooxidative transition metals, and the modification of the physical properties of food (Wang et al. 2016). Carotenoid is regarded as a crucial dietary supplement to enhance antioxidant capacities (Zhang et al. 2019). Moreover, vitamin E acts as vital antioxidant toward harmful oxidation of biological molecules (Niki and Noguchi 2021).

### The multiple regression analysis

The multiple regression analysis was calculated to illustrate the relationship between the detected heavy metals (Cu, Fe, Cd, Pb) and the biochemical content and DPPH activity of the collected *P. radiata*. All regression equations (Eqs. 3–5) depicted the close interaction of the estimated oyster contents and DPPH activity with heavy metals.

3 $${\text{Cu}}\;=\;87.81-0.28\mathrm{\;Energy}-1.16\mathrm{\;Calories}-0.15\mathrm{\;DPPH}+0.78\mathrm{\;Carotenoid}\;+\;10.66\;\upbeta\;-\;{\text{carotenoid}}+6.9\mathrm{\;Fucoxanthin}\;-\;9.27\mathrm{\;Vit\;E}-9.03\mathrm{\;Vit\;C\;}R\;=\;0.89$$

4 $${\text{Cd}}=82.2-0.16\mathrm{\;Energy}+0.01\mathrm{\;Calories}-0.03\mathrm{\;DPPH}-6.3\mathrm{\;Carotenoid}+2.7\upbeta\;-{\text{carotenoid}}+3.97\mathrm{\;Fucoxanthin}-11.91\mathrm{\;Vit\;E}-6.73\mathrm{\;Vit\;C\;}R\;=\;0.99$$

5 $${\text{Pb}}\;=\;454.07+0.01\mathrm{\;Energy}-0.56\mathrm{\;Calories}-0.27\mathrm{\;DPPH}-3.78\mathrm{\;Carotenoid}+97.53+25.59\upbeta\;-{\text{carotenoid}}+25.59\mathrm{\;Fucoxanthin}-67.48\mathrm{\;Vit\;E}-52.18\mathrm{\;Vit\;C\;}R\;=\;0.98$$

As a result of oxidative stress induced by heavy metal accumulation in bivalve tissues, lipid peroxidation, enzyme inactivation, proteins degradation, and DNA damage may occur. Especially, Cu, Cd, and Pb are poisonous and may negatively alter DNA and enzymatic functions, subsequently interacting with life processes (Azizi et al. 2018). However, in order to survive under these unfavorable conditions, bivalves have evolved defense mechanisms against these free radicals and ROS production, which include adjustments of the metabolic rates and activation of alternative energy production pathways (De Almeida et al. 2007). This is in addition to other mechanisms for the elimination and detoxification of metals via creation of antioxidants including vitamins E and C and flavonoids. These antioxidants aid to defend bivalves from lead-induced oxidative damage, in particular, which have been recognized as one of the key causes of lead-related diseases (Lamidi and Akefe 2017). Moreover, bivalve species frequently utilize their proteins and carbohydrate contents as an adaptive response when ROS are overproduced as illustrated in Eq. 2 (Wang et al. 2016).

## Conclusion

Monitoring the effects of heavy metals on oyster species is essential to comprehending the process of bioaccumulation and the extent to which these metals will be transferred to other higher organisms, particularly humans. The pearl oyster species *Pinctada radiata* in the study sites experienced varying degrees of heavy metals pollution. Our results indicated that this species play a vital role in the uptake of heavy metals and that it acts as an ecological biomarker due to its ability to produce non-enzymatic compounds capable of scavenging the produced reactive oxygen species (ROS). As a result, there is a critical need for rapid assessment of these heavy metals in order to control contaminant levels in oysters. In addition, the present results evidently showed that the effects of metal bioaccumulation on oyster growth and biometric measurements depended mainly on the type of metal present and the specific location under investigation. Also, it clearly revealed a serious risk to human health resulting from oysters’ consumption owing to the presence of high magnitudes of metal contamination, especially with lead. Avoiding oyster total consumption would be difficult to apply; however, cautious measures from authorities and consumers should be taken. Results of this study could serve as a starting point for future assessments of metal contamination in order to inform decision-makers about the environmental situation and help them address potential issues resulting from heavy metal pollution, in addition to preserving the sustainability of oyster species found in the Mediterranean Sea. The present findings could also provide valuable insights into the productivity of oysters, particularly in terms of pearl production. Nevertheless, further research are essential to gain a deeper understanding of the underlying mechanisms governing these interactions between ambient heavy metals and bivalves. Such investigations hold the key to understanding the implications of heavy metals pollution for ecosystem health and human welfare.

## Data Availability

All data generated or analyzed during this study are included in this published article.
